# Beyond Hydrogen Sulfide and Cysteine Metabolism: Reframing Cystathionine γ-Lyase as a Potential Translational Regulator of Hypoxia-Inducible Factor-1α in Clear Cell Ovarian Carcinoma

**DOI:** 10.3390/cells15121106

**Published:** 2026-06-18

**Authors:** Amal M. EL-Naggar

**Affiliations:** Department of Pathology, Faculty of Medicine, Menoufia University, Shibin EL-Kom 32511, Egypt; amal.elnaggar.ae@gmail.com

**Keywords:** CTH, HIF-1α, clear cell ovarian carcinoma, moonlighting functions, translation regulation, RNA-binding proteins, cysteine biosynthesis, hydrogen sulfide

## Abstract

**Highlights:**

**What are the main findings?**

- Canonical sulfur metabolism and H_2_S production may not fully explain the diverse biological effects of cystathionine γ-lyase (CTH), including regulation of hypoxia-inducible factor-1α (HIF-1α) expression.
- CTH exhibits multi-compartment localization and structural flexibility and associates with RNA-binding proteins (RBPs) and components of the translational machinery, consistent with broader non-canonical functions.

**What are the implications of the main findings?**

- CTH modulates signaling pathways including PI3K/AKT/mTOR that are linked to translational control and protein synthesis.
- Integrated evidence supports a potential role for CTH in mRNA translation.

**Abstract:**

The canonical transsulfuration (TSS) pathway enzymes cystathionine β-synthase (CBS) and cystathionine γ-lyase (CTH) are traditionally recognized for their roles in the sequential conversion of homocysteine to cysteine and in endogenous hydrogen sulfide (H_2_S) production. Increasing evidence, however, suggests that these enzymes may also exhibit non-canonical (“moonlighting”) functions that extend beyond metabolic regulation. In this review, we evaluate the hypothesis that CTH may participate in translational regulation, particularly in the control of hypoxia-inducible factor-1α (HIF-1α) expression in clear cell ovarian carcinoma (CCOC). We first highlight limitations of the prevailing H_2_S- and cysteine-centric view of the TSS pathway, which may not fully explain emerging context-dependent functions of CTH in cancer biology. Current evidence suggests that CTH enhances HIF-1α protein expression through mechanisms independent of transcription, protein stability, or H_2_S production, implicating a potential role in translational regulation, although direct mechanistic evidence remains limited. To critically evaluate this emerging hypothesis, we categorize evidence according to its level of experimental support, ranging from direct experimental evidence to indirect mechanistic observations and computational predictions. Within this framework, we examine three non-mutually exclusive models: (1) regulation through PI3K/AKT/mTOR-dependent translational signaling; (2) modulation of translational control through interaction with translation-associated proteins and RNA-binding proteins (RBPs) involved in *HIF1A* mRNA regulation; and (3) the more speculative possibility of direct interaction between CTH and *HIF1A* mRNA. Collectively, these observations support a model in which CTH contributes to selective translational regulation beyond its canonical metabolic functions, potentially linking sulfur metabolism to stress-adaptive gene expression in cancer.

## 1. Introduction

Metabolic enzymes are increasingly recognized as multifunctional proteins that extend beyond their classical catalytic roles in cellular metabolism. In addition to regulating metabolic flux, many exhibit non-canonical or “moonlighting” functions, mediated by changes in subcellular localization, post-translational modifications, or protein interactions. Through these mechanisms, metabolic enzymes can function as scaffolds, RNA-binding proteins (RBPs), and regulators of transcriptional or translational processes [1,2]. Importantly, such non-canonical functions are often highly context-dependent, emerging under specific physiological or stress conditions that alter protein localization, interaction networks, or structural states [1].

Protein–protein interactions (PPIs) represent a central mechanism through which proteins acquire such multifunctionality. Through dynamic and context-dependent interactions, proteins can be repurposed to support regulatory functions that are not predicted by their canonical activity [3]. For example, the transcription factor NF-κB recruits ribosomal protein S3 (RPS3) as a non-canonical subunit to enhance DNA binding at specific target genes [4], illustrating how protein–protein interactions can repurpose proteins for regulatory functions.

Beyond PPIs, the identification of RNA-binding capabilities in numerous metabolic enzymes—including GAPDH, PKM2, ASS1, and ADK—has further expanded the concept of protein multifunctionality, revealing that metabolic enzymes can directly participate in post-transcriptional regulation and feedback control of gene expression, thereby adding another layer of complexity to cellular and disease processes, including cancer, reviewed in [5].

The transsulfuration (TSS) pathway represents a metabolic system in which such multifunctionality may be particularly relevant. While traditionally associated with sulfur metabolism and redox homeostasis [6], emerging evidence suggests that enzymes within this pathway may participate in regulatory processes beyond their canonical biochemical roles, including functions related to post-transcriptional gene regulation [7]. However, the extent to which TSS enzymes contribute to these processes remains poorly defined.

In this review, we focus on cystathionine γ-lyase (CTH), a key enzyme of the TSS pathway, and examine evidence suggesting functions beyond its established roles in cysteine metabolism and hydrogen sulfide (H_2_S) production. Particular emphasis is placed on emerging links between CTH and translational regulation, including its potential role in regulating HIF-1α expression in clear cell ovarian carcinoma (CCOC) [7].

## 2. Canonical Functions of CTH and the TSS Pathway

**Figure 1:**
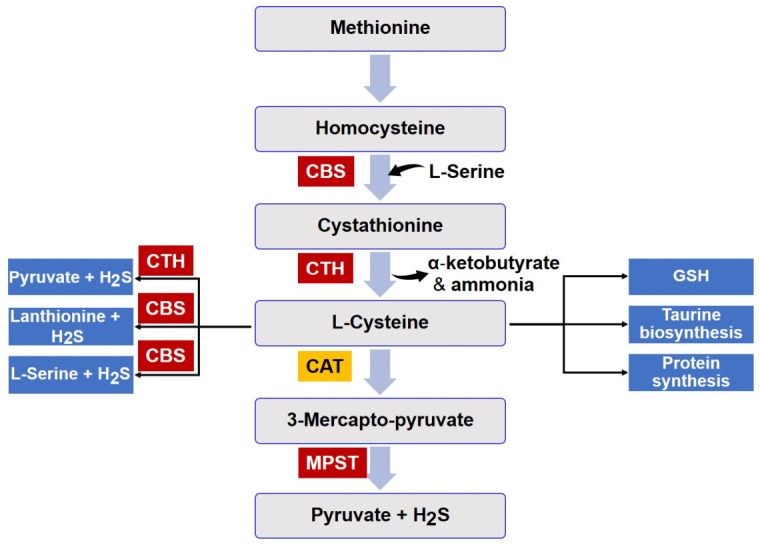
Schematic of the transsulfuration (TSS) pathway. The core pathway is mediated by two pyridoxal-5′-phosphate-dependent enzymes—cystathionine β-synthase (CBS) and cystathionine γ-lyase (CTH)—which convert homocysteine to cysteine, linking methionine metabolism to cysteine biosynthesis. CBS condenses homocysteine with serine to form cystathionine, which is subsequently cleaved by CTH to generate cysteine. Beyond its role in protein synthesis, cysteine serves as a key metabolite in multiple biological processes. Hydrogen sulfide (H_2_S) is generated through multiple enzymatic pathways involving CBS and CTH, as well as 3-mercaptopyruvate sulfurtransferase (MPST) together with cysteine aminotransferase (CAT), reflecting the coordinated contribution of these systems to H_2_S production within the broader sulfur metabolic network.

The TSS pathway is a central component of sulfur metabolism that links methionine metabolism to cysteine biosynthesis while generating key bioactive molecules. It produces cysteine, which serves as a precursor for glutathione (GSH) and supports cellular redox homeostasis, as well as H_2_S, a gaseous signaling molecule with diverse roles in physiological and pathological processes [8,9,10]. This pathway is primarily mediated by the pyridoxal-5′-phosphate-dependent enzymes cystathionine β-synthase (CBS) and cystathionine γ-lyase (CTH, also known as CSE or CGL), which catalyze the conversion of homocysteine to cysteine. Canonically, CBS condenses homocysteine with serine to form cystathionine, which is subsequently cleaved by CTH to yield cysteine. Beyond these canonical reactions, both CBS and CTH can also utilize cysteine and/or homocysteine in β- or γ-elimination reactions that release H_2_S, making sulfide production an intrinsic output of the TSS pathway [10,11]. In addition, 3-mercaptopyruvate sulfurtransferase (MPST) contributes to H_2_S production within the broader sulfur metabolic network [10,12] (Figure 1).

A considerable body of evidence indicates that H_2_S and its primary producing enzymes—CBS, CTH, and MPST— play important roles in cancer progression by promoting tumor cell proliferation, supporting cellular bioenergetics, and stimulating angiogenesis [13,14,15,16]. While the catalytic activities of TSS enzymes and regulation by metabolites such as homocysteine and cysteine are well characterized, increasing evidence suggests additional non-catalytic and regulatory functions. These activities extend beyond canonical roles in cysteine biosynthesis and H_2_S generation. For instance, CBS contains a unique N-terminal heme-binding domain in which the heme is not required for catalytic activity but instead functions as a redox sensor that regulates both CBS enzymatic activity and broader cellular processes [17,18]. Moreover, CBS interacts with the core circadian protein cryptochrome 1 (CRY1) in U2-OS and NIH3T3 cells, implicating it in circadian regulation [19]. In addition, MPST has been shown to regulate inflammatory and epithelial homeostasis in an H_2_S-independent manner through direct interaction with AKT and modulation of its phosphorylation status [20]. Despite these findings, systematic characterization of the non-canonical functions of TSS enzymes remains limited. Notably, CTH is of particular interest because it occupies a central position in sulfur metabolism as the primary mammalian enzyme responsible for de novo cysteine synthesis from cystathionine, thereby supporting glutathione (GSH) production, especially under conditions of limited exogenous cysteine availability [6,15], and as a major source of H_2_S in peripheral tissues [21], with emerging evidence also supporting important roles in the central nervous system [22].

**Figure 2:**
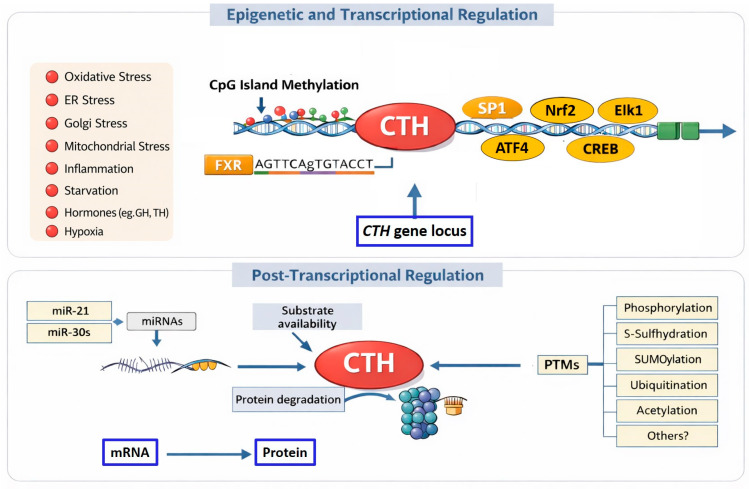
Multilevel regulation of CTH. CTH expression and activity are regulated at epigenetic, transcriptional, and post-transcriptional levels. (Top panel: Epigenetic and transcriptional regulation) *CTH* transcription is modulated by diverse cellular stress signals, including hypoxia, oxidative stress, endoplasmic reticulum (ER) stress, Golgi stress, and mitochondrial stress, as well as inflammation, nutrient deprivation, and hormonal cues (e.g., growth hormone (GH) and thyroid hormone (TH)). Epigenetic regulation via CpG island methylation within the *CTH* promoter represses gene expression. Multiple transcription factors directly bind the *CTH* promoter to regulate transcription: specificity protein 1 (SP1) maintains basal expression; nuclear factor erythroid 2-related factor 2 (Nrf2) mediates induction under oxidative stress; activating transcription factor 4 (ATF4) regulates expression during amino acid starvation; ETS Like-1 protein (Elk1) contributes to promoter activation; and cAMP response element-binding protein (CREB) enhances transcription. In addition, farnesoid X receptor (FXR) directly binds a specific response element (AGTTCAgTGTACCT) within the *CTH* promoter to regulate its expression. (Bottom panel: Post-transcriptional regulation) CTH expression is further controlled by microRNAs (e.g., miR-21 and miR-30s family), which modulate mRNA stability and translation. Protein abundance and enzymatic activity are influenced by substrate availability and protein degradation pathways. Post-translational modifications (PTMs), including phosphorylation, S-sulfhydration, SUMOylation, ubiquitination, and acetylation, as reported in the literature [31,32] and curated in PhosphoSitePlus (www.phosphosite.org, accessed on 13 February 2026), dynamically regulate CTH stability and function. Together, these multilayered regulatory mechanisms coordinate CTH-dependent hydrogen sulfide (H_2_S) production and transsulfuration pathway activity under physiological and pathological conditions. Some elements of the schematic were generated with assistance from ChatGPT (OpenAI, GPT-5).

CTH is a tetrameric enzyme, with each monomer covalently bound to a pyridoxal 5′-phosphate (PLP) cofactor, the active form of vitamin B6. Consistent with its central metabolic and signaling roles, CTH is tightly regulated, and its expression and activity are highly context-dependent. Epigenetic, transcriptional, and post-transcriptional mechanisms collectively modulate CTH expression [10,15,23,24] (Figure 2).

Loss of CTH function causes the rare metabolic disorder cystathioninemia (or cystathioninuria) [25]. While vitamin B6-responsive and nonresponsive forms have been described [26,27], the condition is generally considered clinically mild, and individuals lacking functional enzyme often exhibit favorable outcomes, suggesting that CTH deficiency is well tolerated in humans and is not associated with severe systemic dysfunction [28,29,30].

Studies using *Cth*^−^/^−^ mouse models have yielded variable results. While some reports suggest a role for CTH in neuroprotection, its deficiency has been associated with cognitive impairment [22], and others describe largely normal phenotypes aside from hypertension [33]. Moreover, additional studies have demonstrated that *Cth*^−^/^−^ mice, despite developing normally, exhibit increased susceptibility to oxidative stress under cysteine-restricted conditions [34]. Collectively, these discrepancies likely reflect differences in genetic background, experimental conditions, and species-specific compensatory mechanisms between mice and humans.

In addition to these context-dependent effects observed in loss-of-function models, emerging evidence suggests context-specific non-canonical functions of CTH in distinct cellular settings. Notably, CTH overexpression in CD8^+^ T cells enhances antitumor activity by reshaping the tumor microenvironment through depletion of extracellular glycine, serine, and proline, rather than by promoting T cell proliferation. Although the TSS pathway is present in T cells, CTH overexpression does not confer cysteine independence or rescue proliferation under cysteine/cystine-deprived conditions [35]. Additionally, evidence for non-canonical CTH functions has emerged in neurological disease context. In a Parkinson’s disease-related astrocyte model, increased CTH/CSE expression was associated with astrocyte state transitions towards a neurotoxic phenotype, alongside an overall reduction in H_2_S levels caused by decreased CBS and MPST expression. This effect on astrocytes was linked to FOXD3-mediated transcriptional reprogramming [36], supporting context-dependent regulatory interactions of CTH beyond its canonical role in H_2_S production.

In cancer, increased CTH expression has been associated with cancer progression and metastasis in multiple cancers, including breast cancer [37], prostate cancer [38], glioblastoma [39], nasopharyngeal carcinoma [40], and other malignancies, where the pro-tumorigenic effects of CTH have largely been attributed to activation of the CTH/H_2_S axis; however, direct phenotypic rescue by exogenous H_2_S at physiologically relevant concentrations has not consistently been demonstrated, leaving the specific contribution of H_2_S to these effects incompletely resolved.

Functional studies in specific tumor contexts further challenge an H_2_S-centric model. In Ewing sarcoma, CTH supports survival under cystine limitation by maintaining glutathione-dependent redox homeostasis, and viability can be restored in *CTH* knockdown cells by antioxidant supplementation but not by H_2_S donors [41]. Together, these findings point to context-dependent functions of CTH that are not universally explained by H_2_S production and may extend beyond canonical redox metabolism.

## 3. Limitations of the Canonical Model

### 3.1. Limitations of an H_2_S-Centric View of CTH Function

H_2_S is now recognized as an important gasotransmitter generated through enzymatic and non-enzymatic pathways, as well as from dietary sources and the gut microbiota [8,42]. It interacts with diverse biomolecules and regulates processes including metabolism, vascular tone, immune responses, and neuronal signaling with potential implications for both health and disease [8,43].

Early investigations into H_2_S biology were shaped by its established toxicity as an environmental hazard [44,45], driven by its potent inhibition of cellular respiration, later attributed to the suppression of cytochrome c oxidase (complex IV) in the mitochondrial electron transport chain [46,47,48]. This toxicological view was further reinforced by early experimental studies employing supraphysiological (high micromolar to millimolar) concentrations of fast-releasing inorganic donors (e.g., Na_2_S or NaHS) [49,50]. While earlier studies were instrumental in defining the chemical reactivity of H_2_S, the concentrations used often exceed those found in vivo and may not fully reflect endogenous signaling conditions. Consequently, these approaches may have disproportionately emphasized inhibitory effects that differ from those occurring under physiological conditions. High donor concentrations likely overwhelmed redox-sensitive targets, eliminated signaling specificity, and induced acute mitochondrial inhibition that dominated cellular phenotypes. Such conditions may also have obscured dose-dependent signaling effects, reinforcing the misconception that H_2_S functions primarily as a toxic or metabolic inhibitor (see reviews [51,52,53,54]).

More recent quantitative analyses indicate that free H_2_S levels in tissues are typically maintained within the low nanomolar to low micromolar range, with rapid turnover and tightly regulated spatial dynamics [55,56]. Within this physiological window, H_2_S exerts context- and compartment-dependent effects on mitochondrial function and cellular bioenergetics and engages signaling mechanisms distinct from those observed at higher concentrations, potentially including protein persulfidation of reactive cysteine residues or signaling via reactive sulfur species such as polysulfides [52,53,54,57]. Collectively, these findings support a model in which H_2_S functions as a regulated signaling molecule whose biological effects are highly dependent on concentration, cellular context, and subcellular localization.

In cancer, H_2_S can exert context-dependent and sometimes opposing effects depending on tumor type, metabolic state, and microenvironment, displaying both tumor-promoting activities, as reported in NSCLC [58], esophageal cancer [59], colorectal cancer [60], among others, and tumor-suppressive effects, as observed in the ID8 murine epithelial ovarian cancer cell line [61] and in human breast adenocarcinoma as well as hepatocellular carcinoma cell lines [62] (see also the review in [63]).

Experimental studies using exogenous H_2_S donors further support the idea that sulfide signaling produces distinct effects across cellular systems. Lee et al. [64] showed that H_2_S donors (GYY4137 and NaHS) induce G2/M cell cycle arrest and apoptosis across multiple cancer cell lines, with limited effects in normal fibroblasts. Similarly, Xiao et al. [65] reported that Na_2_S increases reactive oxygen species (ROS) levels in glioblastoma cells, promoting apoptosis and enhancing radiosensitivity, effects not observed in normal human brain microvascular endothelial cells. In line with these findings, Zhao et al. [66] demonstrated that NaHS induces apoptosis in glioma cells through activation of p38/MAPK and p53 signaling pathways. Collectively, these studies highlight the context-dependent nature of H_2_S signaling across cellular systems.

CTH has historically been considered a major enzymatic source of H_2_S, supported by its tissue expression profile and murine knockout studies showing reduced H_2_S levels upon genetic deletion [33,67,68]. However, this model is increasingly challenged by human genetic and metabolic data. Kozich et al. demonstrated that in patients with rare inborn errors affecting CBS or CTH, circulating bioavailable sulfide is not decreased and may even increase in CBS deficiency, indicating strong compensatory mechanisms that preserve systemic sulfur homeostasis [69]. These findings are particularly relevant for cancer biology, where enzymatic dependencies inferred from preclinical models may not directly reflect human metabolic adaptation. Instead, they suggest that disruption of canonical TSS enzymes may be buffered by alternative enzymatic, nutritional, or microbiota-derived sulfur sources.

Further supporting this concept, recent studies in mammalian systems, including mouse models, show that sulfide and persulfide production can persist even when canonical TSS enzymes are disrupted, owing to the activity of cysteinyl-tRNA synthetase (CARS), which functions as a cysteine persulfide synthase and contributes substantially to intracellular sulfide and persulfide production in mammals [70]; these species are also proposed to serve as a reservoir for H_2_S [71]. Of interest, earlier work suggested that CBS- and CTH-mediated reactions predominantly generate persulfides rather than free H_2_S, with H_2_S largely arising as a downstream product of persulfide degradation [72]. This view has since evolved with evidence that persulfides function as distinct bioactive sulfur species rather than merely intermediates in H_2_S release [73]. Recent work further indicates that their signaling properties are chemically diverse and may involve electrophilic redox signaling and direct sulfur transfer through transpersulfidation reactions independent of free H_2_S generation [74]. These findings further highlight that persulfide biology cannot be reduced to H_2_S release alone, but involves direct and chemically diverse modes of action. Consistent with this buffered and distributed sulfur network, endothelial-specific deletion of CTH in mice does not significantly alter tissue H_2_S levels but instead affects sulfane sulfur pools [75], indicating that H_2_S output is maintained through metabolic redundancy and that sulfur regulation extends beyond single-enzyme control.

Beyond sulfur buffering, several studies also suggest that CTH-associated phenotypes may diverge from canonical H_2_S output. Geng et al. reported that both pharmacological elevation of H_2_S levels using a donor and inhibition of endogenous H_2_S production through CTH blockade improved insulin sensitivity in obese mice, despite exerting opposite effects on lipolysis [76]. These findings suggest that CTH-associated metabolic effects are not strictly concordant with H_2_S signaling and may reflect partially decoupled roles in metabolic regulation.

Furthermore, Bibli et al. showed that inflammatory signaling induces phosphorylation of CTH at Ser377, resulting in enzymatic inactivation and reduced H_2_S production despite maintained or increased CTH protein expression [77]. This indicates that post-translational modification can uncouple CTH abundance from H_2_S production, suggesting that CTH activity—and its downstream functions—may be regulated independently of canonical sulfide output. Importantly, enzyme-selective regulation of H_2_S production has also been reported, as Qi et al. demonstrated that estrogen-dependent H_2_S production in endometrial stromal cells is mediated specifically through CBS rather than CTH, supporting enzyme-selective regulation of H_2_S signaling in a physiological context [78].

Collectively, these observations challenge the assumption that CTH-derived H_2_S, and potentially H_2_S produced by other enzymes, represents a stable or direct functional output across biological contexts. Instead, they suggest that H_2_S may not fully account for CTH-associated biological activity, and support a model in which tightly regulated sulfide levels operate as part of a broader network of reactive sulfur species and metabolic fluxes.

### 3.2. Limitations of a Cysteine-Centric View of CTH Function

Cysteine metabolism highlights a parallel limitation of the canonical model. Cysteine is central to redox homeostasis and anabolic metabolism, serving as a precursor for glutathione (GSH), iron–sulfur clusters, coenzyme A, and other sulfur-containing metabolites, while also contributing directly to protein synthesis [6,79]. Although TSS-derived cysteine production has been proposed to support survival under cysteine-limiting conditions in certain cancers such as neuroblastoma [13,80] and Ewing sarcoma [41], its importance is highly context-dependent and not universally rate-limiting across cancers. In line with this, Zhang et al. found that CBS and CTH expression levels do not correlate with cysteine starvation sensitivity across cancers, which instead depends on broader metabolic context, including polyamine-associated metabolic rewiring that increases oxidative stress under cysteine limitation [81].

Functional studies further support the limited buffering potential of TSS under nutrient stress. Kang et al. showed that in a broad panel of non-small cell lung cancer (NSCLC) cell lines, de novo cysteine synthesis is insufficient to sustain intracellular cysteine pools during cystine starvation, leading to impaired GSH synthesis and ferroptosis, an oxidative stress-driven form of cell death. Although partial compensation occurs through glutamate–cysteine ligase catalytic subunit (GCLC)-mediated γ-glutamyl peptide production, this mechanism primarily limits glutamate accumulation rather than restoring canonical antioxidant capacity [82]. Extending these observations to hematological malignancies, studies in acute myeloid leukemia (AML) showed that CRISPR-mediated CTH loss did not substantially sensitize cells to cystine deprivation, although differences in CBS and CTH expression influenced the ability of AML cells to adapt to cysteine stress [83]. In vivo metabolic tracing studies corroborate these findings, demonstrating that TSS contributes minimally to cysteine pools in most non-hepatic tissues, where extracellular cyst(e)ine remains the dominant source. Tumor cysteine metabolism therefore largely reflects tissue-of-origin constraints rather than intrinsic TSS capacity [84].

### 3.3. Evidence for Non-Canonical CTH Function in Clear Cell Ovarian Carcinoma (CCOC)

CCOC may represent a useful model system for investigating the TSS, as it exhibits a functional TSS pathway, relies on cysteine metabolism for growth, and displays heterogeneous sensitivity to cysteine deprivation across cell lines. Cysteine deprivation is further associated with context-dependent oxidative stress responses, including necrosis, ferroptosis, and apoptosis depending on metabolic state. Notably, intracellular cysteine levels vary across cell lines, whereas glutathione levels remain comparable despite differences in basal cysteine abundance [85], suggesting that cysteine availability may not be directly reflected in steady-state glutathione pools. Furthermore, ferroptosis sensitivity is influenced not only by sulfur metabolism but also by oncogenic signaling. Hyperactivation of the PI3K/AKT/mTORC1 pathway promotes ferroptosis resistance through lipid remodeling programs involving SREBP1 and the accumulation of monounsaturated fatty acids, potentially mediated by SCD1 [86,87]. This is particularly relevant for CCOC, which frequently harbors PI3K/AKT pathway activation, suggesting that ferroptosis resistance may arise from the integration of metabolic, lipid, and signaling networks rather than isolated metabolic inputs or redox pathways alone.

CCOC also provides a compelling model for examining potential non-canonical functions of CTH. We previously demonstrated that CTH, but not CBS or MPST, is required for hypoxia-induced HIF-1α protein accumulation in CCOC cells [7], indicating a CTH-specific effect. At the transcriptional level, *HIF1A* mRNA levels in *CTH* knockout (KO) cells were comparable to control cells, and actinomycin D-based mRNA decay assays showed no difference in *HIF1A* transcript stability, arguing against transcriptional regulation. At the protein level, neither the proteasome inhibitor MG132 nor the prolyl hydroxylase inhibitor DMOG fully restored HIF-1α in *CTH* KO cells to levels comparable to those in control cells, and cycloheximide chase experiments showed similar HIF-1α degradation rates between conditions. Together, these data exclude altered degradation or protein stability as mechanisms [7].

Given known links between CTH and H_2_S, our previous study observed increased H_2_S levels in *CTH* KO cells, likely due to compensatory CBS upregulation and enhanced CBS activity, both of which were experimentally demonstrated [7]. This was particularly relevant in light of prior studies demonstrating that H_2_S can regulate HIF-1α in a context-dependent manner, with reports of both inhibitory [88] and stimulatory [89] effects depending on cellular system and experimental conditions, and that CBS-derived H_2_S can regulate HIF-1α stability in non-ovarian models through persulfidation and activation of prolyl hydroxylase 2 (PHD2), thereby promoting HIF-1α hydroxylation and degradation [90]. However, several observations argued against H_2_S-mediated regulation in the CCOC model. Pharmacologic supplementation with either the slow-releasing H_2_S donor GYY4137 or the fast-releasing donor Na_2_S failed to alter HIF-1α protein expression in control cells. Furthermore, a catalytically impaired CTH mutant carrying an arginine-to-alanine substitution at residue 62 (R62A), a residue reported to be essential for CTH enzymatic activity [91], restored HIF-1α expression in *CTH* KO cells, supporting a potential non-enzymatic role for CTH. In addition, CTH-driven phenotypes, including reduced viability and motility, are not fully explained by redox metabolism, as antioxidant supplementation or glutathione monoethyl ester (GSH-MEE) fails to fully rescue the effects of CTH loss under cysteine-replete conditions [7]. Together, these findings indicate that CTH-dependent regulation of HIF-1α cannot be fully explained by established roles in sulfur metabolism, redox control, or H_2_S production.

Collectively, these observations across Section 3.1, Section 3.2 and Section 3.3 support a conceptual shift in which sulfur metabolism in mammalian systems is better described as a buffered, redundant, context-dependent network of reactive sulfur species and metabolic fluxes rather than linear outputs of H_2_S or cysteine production. Within this framework, CTH may function as a node in this distributed regulatory system contributing to redox balance, metabolic plasticity, and stress adaptation. In cancer, the frequent upregulation of CTH may reflect integration into broader metabolic and stress-response programs, alongside additional non-canonical functions beyond its enzymatic roles that remain to be defined. Structural and spatial mechanisms may further contribute to these non-canonical functions.

## 4. Spatial and Structural Basis for CTH Non-Canonical Functions

CTH is classically reported as a predominantly cytosolic enzyme [92]; however, multiple studies suggest that it may localize to diverse intracellular and extracellular compartments, including the nucleus [93], mitochondria [94], plasma membrane [95], endoplasmic reticulum [95], plasma [96], and urine [97], where it may exert compartment-specific functions. In addition, large-scale proteomic analyses [98], sequence-based predictions, and corresponding annotations in curated databases such as GeneCards [99] support a multi-compartment distribution of CTH across intracellular and extracellular environments (Table 1), warranting further investigation of its context-specific functions.

Post-translational modifications (PTMs) may provide a mechanistic basis for such dynamic subcellular distribution. Agrawal et al. demonstrated that both CBS and CTH can undergo SUMOylation in vitro, a modification proposed to regulate nuclear translocation and potentially enable compartment-specific functions [31]. Consistent with this, Drekolia et al. [93] recently demonstrated that CTH can translocate to the nucleus in a cystine-dependent manner despite lacking a classical nuclear localization signal, where it contributes to cystine oxidation and the generation of acetyl units that promote histone H3 acetylation and chromatin remodeling, thereby linking metabolism to transcriptional regulation.

In addition, both CTH and CBS are secreted by endothelial cells and hepatocytes, circulate in plasma, and remain catalytically active in blood, generating H_2_S from homocysteine [96]. This extracellular activity further indicates that TSS enzymes are not confined to intracellular metabolism. Collectively, these observations support a broader model in which CTH operates across intracellular and extracellular environments rather than as a solely cytosolic enzyme in cysteine metabolism and H_2_S generation. This spatial distribution may enable context-dependent functions through both catalytic and interaction-driven mechanisms.

Beyond spatial compartmentalization, intrinsic structural properties of CTH may further contribute to its functional plasticity. Structural studies of human CTH reveal that the apo enzyme adopts an open conformation in the absence of PLP, transitioning to a more closed state upon cofactor binding. This ligand-dependent conformational shift highlights the intrinsic flexibility of the protein [100], suggesting that CTH can sample multiple structural states. Such conformational plasticity may enable interactions beyond its canonical catalytic role, including context-dependent non-canonical functions.

**Figure 3:**
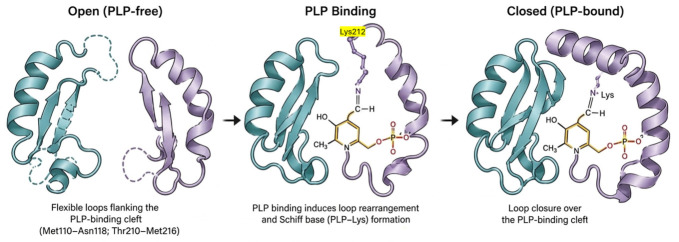
Proposed model for cofactor-dependent conformational changes in CTH. In the absence of pyridoxal 5′-phosphate (PLP), CTH adopts an open conformation characterized by increased flexibility in loop regions flanking the active site, including Met110-Asn118 and Thr210-Met216. PLP binding induces local ordering and formation of a Schiff base linkage with the catalytic residue Lys212, stabilizing interactions within the active site and promoting a transition to a closed, catalytically competent conformation. This ligand-dependent conformational switch reflects intrinsic structural plasticity of the enzyme and enables sampling of multiple functional states. The schematic depicts a simplified dimeric unit for visual clarity, where each color denotes an individual monomer; the functional enzyme exists as a homotetramer, with one PLP molecule bound per monomer. Some elements of the schematic were generated with assistance from ChatGPT (OpenAI, GPT-5).

In line with this conformational plasticity, additional structural analyses indicate that CTH is not rigidly folded but exhibits localized flexibility and partial disorder in specific regions [91]. Notably, two loop regions (Met110-Asn118 and Thr210-Met216), which flank the PLP-binding cleft, undergo conformational rearrangement upon cofactor binding, folding back over the active site to stabilize ligand interactions (Figure 3). Although these observations do not indicate PLP-independent catalytic activity, they suggest that CTH structure is sensitive to its molecular environment, a property that may facilitate regulated interactions or non-canonical functions in specific cellular contexts. Such structural plasticity is a recognized feature of proteins capable of transient and multivalent interactions.

**Figure 4:**
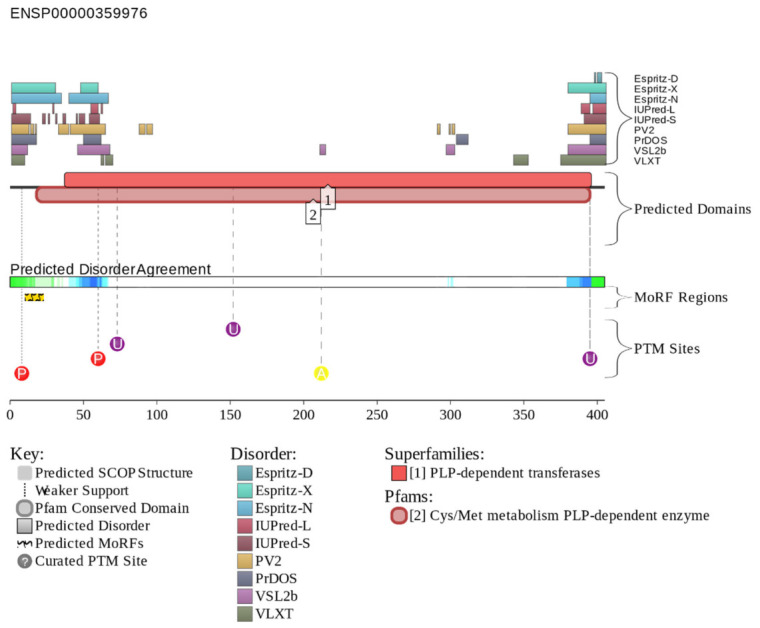
Intrinsic disorder landscape of human CTH. Intrinsic disorder predictions for human CTH (Ensembl ID: ENSP00000359976; UniProt ID P32929) were retrieved from the D2P2 database (https://d2p2.pro/search, accessed on 24 March 2026). Outputs from nine specialized disorder prediction algorithms—Espritz-D, Espritz-X, Espritz-N, IUPred-L, IUPred-S, PV2, PrDOS, VSL2b, and VLXT—are mapped as stacked, pastel-colored tracks along the amino acid sequence. The Predicted Disorder Agreement bar displays the consensus gradient among all nine predictors, highlighting regions of high and low disorder agreement across the protein sequence. Predicted Structural Classification of Proteins (SCOP) structural superfamilies are displayed as bright rounded blocks, identifying the (1) PLP-dependent transferase superfamily and (2) Cys/Met metabolism PLP-dependent enzyme Pfam family. A molecular recognition feature (MoRF region) is localized within the disordered segments. Documented post-translational modification (PTM) sites are aligned at the bottom, noting specific coordinates for curated phosphorylation (P), ubiquitination (U), and acetylation (A) events. Abbreviations: SCOP, Structural Classification of Proteins; Pfam, Protein Families database; MoRFs, molecular recognition features; PTM, post-translational modification.

Partial disorder and conformational flexibility are increasingly recognized as enabling features for nucleic acid interactions. Although classical RBPs typically contain defined RNA recognition motifs (RRMs) or KH domains, many non-canonical RBPs lack these canonical features yet still associate with RNA [101,102,103]. Intrinsically disordered regions (IDRs) play a key role in mediating such interactions by enabling conformational adaptability and dynamic binding, and are increasingly recognized as hallmark features of proteins that function as interaction hubs within signaling networks [104,105,106,107]. In this context, CTH exhibits features consistent with RNA-binding potential, which may contribute to its capacity to engage diverse interaction partners. For example, its N-terminal region includes an unstructured segment, and specific regions flanking the PLP-binding cleft can show flexibility and can become partially disordered under certain conditions [91]. Computational analysis using the Database of Disordered Protein Predictions (D2P2) [108] (https://d2p2.pro/search, accessed on 24 March 2026), by querying human CTH UniProt ID P32929, predicts multiple intrinsically disordered regions in human CTH, some of which overlap with potential post-translational modification sites (Figure 4), suggesting regulatory complexity. Consistent with this structural disorder, *Pseudomonas aeruginosa* CTH/CGL (PaCGL) shows similar features, including an unstructured N-terminal segment (first 13 residues), a disordered loop region (residues 46–57) [109], and localized conformational variability in two long loops (L23–60 and L347–370), which can adopt partially disordered or alternative conformations, including an extended non-helical state in one subunit [109]. Together, these structural and spatial properties may provide a mechanistic basis for the involvement of CTH in diverse molecular interaction networks.

## 5. CTH Interaction Networks and Positioning in Regulatory Systems

Building on its multi-compartment distribution, CTH engages in protein interaction networks that may underlie its context-dependent functions. Emerging evidence suggests that these interactions extend beyond canonical metabolic roles, implicating CTH in signaling processes, stress responses, and transcriptional regulation. Hu et al. showed that CTH counteracts endothelial cell senescence by binding to p53, promoting its cytoplasmic retention and preventing its acetylation and transcriptional activation, independently of p53 S-sulfhydration by H_2_S [110]. Furthermore, Zhu et al. demonstrated that CTH forms a complex with YAP in a Parkinson’s disease-related astrocyte model, facilitating FOXD3-mediated transcriptional regulation while modulating YAP nuclear translocation [36]. In line with this emerging view of CTH as a multifunctional regulatory protein, Drekolia et al. [93] reported that CTH/CSE associates with pyruvate dehydrogenase E1 subunit alpha 1 (PDHA1) in both cytosolic and nuclear compartments of pre-proliferative endothelial cells in a cystine-dependent manner, with coordinated nuclear localization suggesting compartment-specific metabolic–nuclear coupling. Reciprocal immunoprecipitation further revealed that PDHA1 complexes contain histone acetyltransferases, including GCN5 and HAT1, suggesting the assembly of a nutrient-sensitive multi-protein complex linking metabolic enzymes to chromatin-modifying machinery. Notably, this interaction network is associated with selective regulation of histone H3-, H4-, and H2A-associated acetylation marks, particularly H3K9ac, H3K23ac, and H2AK5ac, accompanied by downstream changes in chromatin accessibility and transcriptional programs. Importantly, Drekolia et al. [93] further showed that perturbation of sulfide flux using a fast-releasing H_2_S donor does not significantly impact vascular growth in vivo, suggesting a limited contribution of H_2_S signaling in this context. Together, these findings support a model in which CTH participates in context-dependent protein interaction networks that integrate metabolic status with signaling and transcriptional regulation.

**Figure 5:**
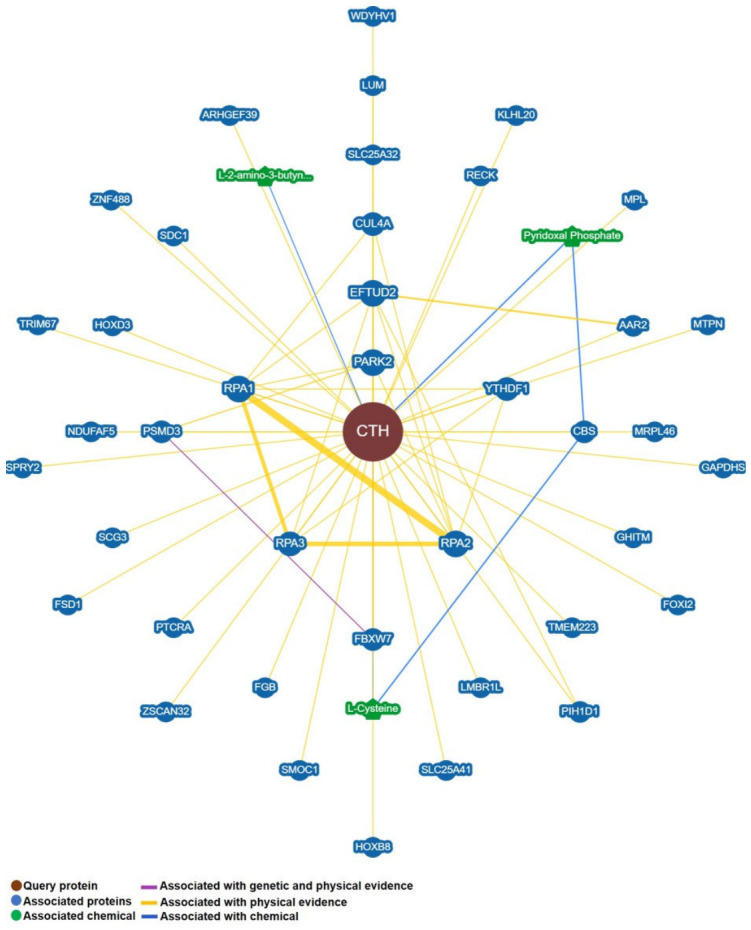
CTH-associated protein network. Curated *Homo sapiens* CTH-associated protein interaction network derived from the BioGRID database (version 5.0.257; https://thebiogrid.org/107873/summary/homo-sapiens/cth.html; accessed on 14 May 2026). The network highlights representative proteins associated with translational initiation, immune, metabolic, transcriptional, and structural pathways. This non-exhaustive subset of CTH-associated proteins illustrates major functional themes emerging from systems-level protein association data. ** Greater node size represents increased connectivity and thicker edge sizes represent increased evidence supporting the association.

At a systems level, curated interaction databases (BioGRID [111,112], IntAct [113,114], HitPredict [115]) and large-scale affinity purification–mass spectrometry (AP-MS)-based proteomic interactome datasets [98,116,117,118,119] consistently place CTH within extensive protein association networks across eukaryotic systems. These resources collectively indicate that CTH participates in broad functional modules spanning immune, metabolic, transcriptional, and structural pathways. To provide a representative overview, Figure 5 summarizes a curated subset of CTH-associated interactions derived from the BioGRID database (version 5.0.257), highlighting representative proteins involved in translational regulation, signaling, and cellular homeostasis. This includes YTHDF1 [120], PARK2 [121], SDC1 [98], CUL4A [122], along with additional network-associated proteins such as RECK, WDYHV1, PTCRA, SMOC1, SLC25A32, HOXD3, KLHL20, ARHGEF39, and SCG3.

Importantly, additional CTH-associated proteins identified through independent interaction studies and curated datasets, including YWHAZ (14-3-3ζ/δ) [123] and the RBPs ELAVL1/HuR and PTBP1 [77], further expand this interaction landscape beyond the BioGRID-derived subset shown in Figure 5. Collectively, these observations position CTH within broader signaling and post-transcriptional regulatory networks potentially linked to processes such as mRNA translation and cellular stress responses. The functional and subcellular diversity of these interacting partners—spanning cytoplasmic, mitochondrial, nuclear, and secretory compartments—is consistent with the reported multi-compartment localization of CTH [98] and supports its activity across diverse cellular contexts. However, functional validation of many of these interactions remains unresolved. Together, these findings support the emerging view that CTH interfaces with multiple signaling and RNA-regulatory networks.

## 6. CTH and Translational Regulation

Emerging evidence places CTH at the interface between cellular signaling pathways and the molecular machinery governing protein synthesis, suggesting it may influence translation through both indirect signaling effects and proximity-based interactions with translational regulators.

### 6.1. Signaling-Level Regulation of mRNA Translation

CTH has been linked to modulation of the PI3K/AKT/mTOR pathway, a central regulator of mRNA translation. In breast cancer models, CTH has been shown to positively regulate the PI3K/AKT pathway, with its upregulation increasing PI3K, Akt, and phospho-Akt levels and its knockdown producing the opposite effect [37]. This is consistent with findings in triple-negative breast cancer (TNBC) cells, where pharmacological inhibition of CTH reduces PI3K/AKT signaling, as evidenced by decreased PI3K and AKT levels as well as AKT phosphorylation in MDA-MB-231 and MDA-MB-468 cells [124]. Similarly, in nasopharyngeal carcinoma models, CTH overexpression increases phospho-PI3K, phospho-AKT, and phospho-mTOR levels, whereas knockdown reduces their expression [40]. Through this signaling axis, CTH may influence downstream translational regulators such as 4E-BPs and S6K, thereby affecting mRNA translation through mechanisms that include modulation of eIF4F complex assembly; however, direct evidence for regulation of these translational effectors by CTH remains lacking. These effects are likely context-dependent and shaped by cellular redox state and metabolic flux.

### 6.2. Association with Translational Machinery Components

Beyond upstream signaling, large-scale interactome studies suggest that CTH is linked to components of the translational machinery identified across species. CTH has been shown to associate with ribosomal protein S2 (RpS2) in *Drosophila melanogaster* and *Saccharomyces cerevisiae* [125], as well as with eIF3j, a subunit of the eukaryotic translation initiation factor 3 complex, in *Drosophila melanogaster* [126]. Given the evolutionary conservation of both CTH and eIF3j, these findings suggest a potential conserved association with translation initiation machinery.

Broader interactome datasets extend this network to additional translational and signaling-associated proteins. CTH-associated proteins include YTHDF1 [120] and YWHAZ (14-3-3ζ/δ) [123]. YTHDF1, a canonical m^6^A reader, promotes translation efficiency of target mRNAs through recruitment of eukaryotic initiation factor 3 (eIF3) [127,128], while YWHAZ has been implicated in selective mRNA translation [123,129], and in PI3K/AKT/mTOR pathway activation [130]. Additional network-level evidence links CTH to PARK2 [121], which has been reported to interact with translation initiation factors including eIF4B in mammalian cells [131,132] and eIF4E in *Drosophila melanogaster* [133]. Furthermore, CTH-associated AP-MS datasets identify interacting partners including SDC1 [98] and CUL4A [122], both of which function as modulators of PI3K/AKT signaling [134,135], thereby providing additional indirect links to mRNA translational control.

### 6.3. RNA-Binding Proteins and Transcript-Selective Regulation

Additional interactome analyses in murine endothelial cells link CTH to RBPs, including ELAVL1 and PTBP1 [77]. Both proteins are established regulators of *HIF1A* mRNA translation [136], suggesting a potential link between CTH-associated complexes and transcript-selective translational control.

### 6.4. Integrated Model

Collectively, these findings support a model in which CTH is positioned within translational regulatory networks through a combination of signaling-mediated effects and protein interaction-dependent associations. This framework suggests involvement of CTH in both global translational control and more selective regulatory processes, although direct mechanistic evidence remains limited and further experimental validation is required.

## 7. CCOC as a Contextual Framework for CTH Non-Canonical Function

**Figure 6:**
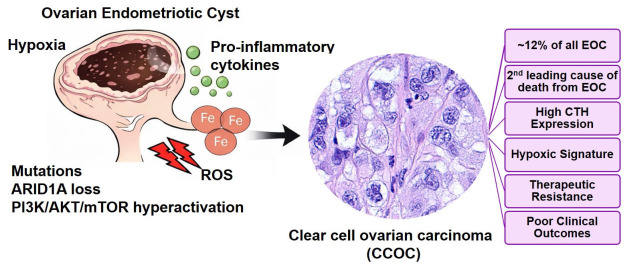
Schematic representation of CCOC development and key molecular features. CCOC is proposed to arise from endometriotic cysts characterized by a pro-inflammatory, iron-rich, and hypoxic microenvironment that promotes the accumulation of ROS and facilitates the acquisition of oncogenic alterations, including loss of ARID1A and activation of the PI3K/AKT/mTOR pathway. These events contribute to tumor initiation and progression. Established CCOC is characterized by a hypoxic signature, elevated CTH protein expression, stress resistance, and chemoresistance, all of which are associated with poor clinical outcomes. Abbreviations: EOC, epithelial ovarian cancer; Fe, iron. Some elements of the schematic were generated with assistance from ChatGPT (OpenAI, GPT-5).

Motivated by findings presented in preceding sections, CCOC provides a biologically and clinically relevant context to further investigate the non-canonical functions of CTH. Although current mechanistic insights in this context are still emerging and are informed by prior studies, including our own [7], CCOC provides a valuable framework for exploring alternative paradigms of CTH biology. CCOC, which often arises from ovarian endometriotic cysts (endometriomas) [137], is an aggressive subtype of epithelial ovarian cancer, accounting for approximately 12% of cases and characterized by limited treatment options and poor clinical outcomes [138,139]. This malignancy is defined by a distinct molecular and metabolic landscape, including frequent loss of ARID1A, hyperactivation of the PI3K/AKT/mTOR pathway, and a pronounced hypoxic signature [138,140,141,142] (Figure 6). In addition, CCOC exhibits marked resistance to oxidative and metabolic stress through adaptive antioxidant mechanisms that extend beyond glutathione-dependent redox homeostasis [143]. Collectively, these features create a cellular environment in which translational control plays a critical role in tumor adaptation and progression.

Notably, CTH expression is elevated in CCOC relative to other epithelial ovarian cancer subtypes [144,145]. In this context, and as discussed in Section 3.3, CTH-dependent regulation of HIF-1α in CCOC is not explained by canonical enzymatic functions or by transcriptional and protein stability mechanisms [7]. Given the central role of translational regulation in controlling HIF-1α protein expression [146,147,148], these observations raise the possibility that CTH may contribute to HIF-1α regulation and stress adaptation through mechanisms potentially linked to translational regulation. Of note, CTH expression is further upregulated in CCOC cells under hypoxia and following treatment with endometriotic cyst contents, consistent with a potential role in adaptive responses to the endometriosis-associated tumor microenvironment [7]. Within this framework, activation of the PI3K/AKT/mTOR pathway in CCOC provides a strong driver of mRNA translation, while hypoxic and oxidative stress conditions favor selective translation of stress-responsive transcripts [149,150]. CTH may therefore function as a modulatory factor integrating metabolic signaling with mRNA translation.

## 8. Conceptual Model: CTH as a Context-Dependent Regulator of mRNA Translation

### 8.1. Model Overview

**Figure 7:**
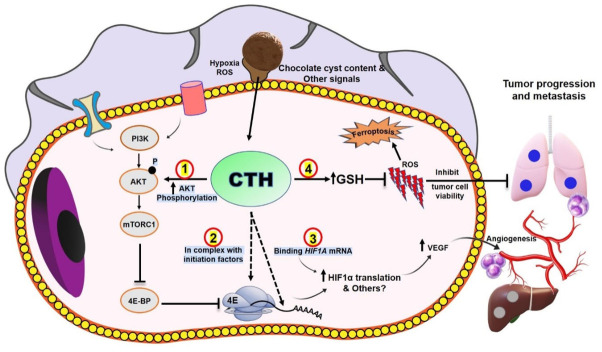
Schematic representation of a conceptual, hypothesis-generating model proposing CTH as a potential context-dependent regulator of mRNA translation in CCOC. In CCOC, CTH is upregulated in response to cellular stress conditions, including hypoxia, ROS, and exposure to endometriotic cyst contents. CTH may influence PI3K/AKT signaling (1) and may associate with components of the translational initiation machinery (2). Beyond these effects, CTH is proposed to interact with *HIF1A* mRNA either directly or indirectly through RBPs, thereby enhancing its translation (3). In parallel, through its canonical role in cysteine metabolism, CTH contributes to GSH-dependent redox homeostasis and protection against ferroptosis (4). These mechanisms are proposed to enhance HIF-1α protein expression and may thereby promote VEGF-mediated angiogenesis, tumor progression, and adaptive stress responses in CCOC.

Based on converging structural, interactome, and functional evidence, we propose a model in which CTH may function as a context-dependent regulator of mRNA translation, including potentially *HIF1A* and other adaptive transcripts, thereby linking cellular metabolic state to adaptive gene expression programs in CCOC. Figure 7 summarizes four regulatory axes through which CTH may influence CCOC progression and adaptation to its microenvironment: (1) modulation of PI3K/AKT signaling, a pathway frequently reported to be altered upon CTH modulation; (2) association with components of the translational initiation machinery; (3) interaction with *HIF1A* mRNA, either directly or indirectly through RBP-mediated mechanisms; and (4) maintenance of glutathione-dependent redox homeostasis and ferroptosis resistance. Collectively, these pathways may promote cellular adaptation to hypoxic and oxidative stress conditions characteristic of the CCOC microenvironment. The first three axes form the basis of the translational regulatory mechanisms discussed below, whereas redox homeostasis represents a parallel adaptive pathway that may cooperate with translational control.

### 8.2. Mechanistic Models of Translational Regulation

CTH function in translational regulation can be mechanistically conceptualized across three complementary and non-mutually exclusive models.

Model 1: CTH may modulate global translational control through the PI3K/AKT/mTOR signaling axis, thereby modulating overall translation capacity. This mechanism is supported most directly by evidence linking CTH perturbation to changes in this pathway across tumor contexts.

Model 2: CTH may contribute to transcript-selective translation through associations with components of the translational machinery in both cross-species systems (e.g., eIF3j and RpS2) and mammalian systems (e.g., YTHDF1, which promotes translation via recruitment of eIF3), as well as RBPs such as the *HIF1A* mRNA translational regulators ELAVL1 and PTBP1. Experimental validation of ELAVL1 [77] supports the RNA-binding protein-associated interactions, although direct evidence linking CTH to selective translational control remains limited.

**Figure 8:**
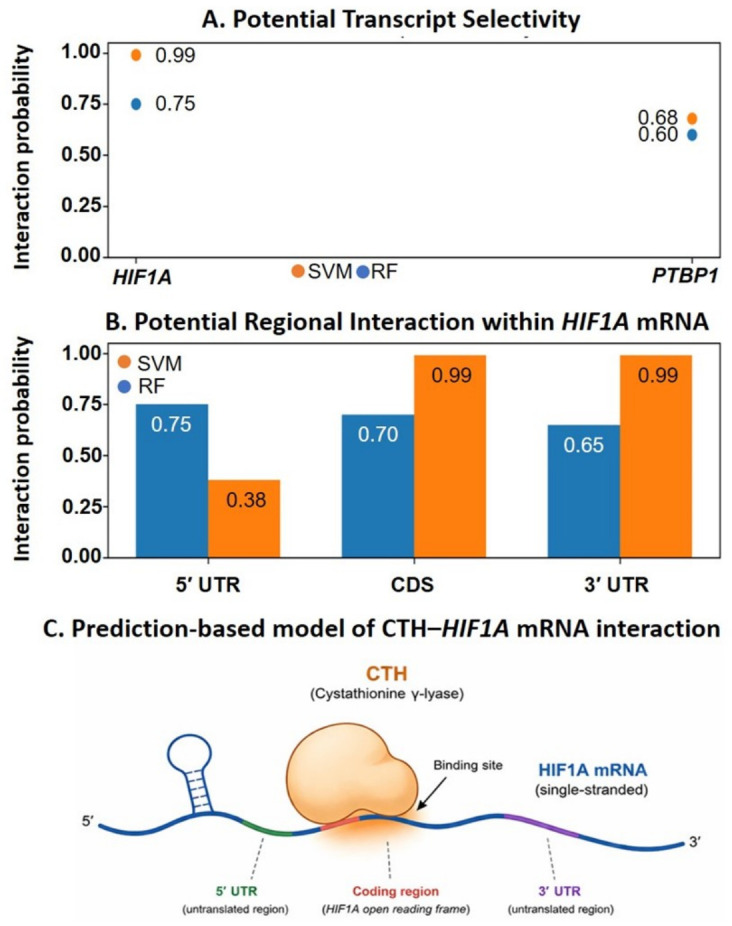
Computational prediction of potential interaction between CTH protein and *HIF1A* mRNA. (**A**) RPISeq prediction scores generated using Random Forest (RF) and Support Vector Machine (SVM) classifiers indicate a higher interaction probability between CTH and *HIF1A* mRNA compared to a comparison transcript (*PTBP1*), suggesting potential transcript selectivity. (**B**) Region-specific analysis across the *HIF1A* transcript reveals heterogeneous interaction probabilities, with stronger predicted binding in the coding sequence (CDS) and 3′UTR relative to the 5′UTR. Values represent classifier probabilities, with scores > 0.5 considered indicative of potential positive RNA–protein interaction. These predictions do not confirm binding, identify binding sites or establish functional interaction and therefore require experimental validation. (**C**) Schematic representation of a proposed, prediction-based model highlighting preferential interaction of CTH within the *HIF1A* coding sequence (CDS). This model is hypothesis-generating and intended to illustrate a testable framework derived from computational prediction; it does not represent experimentally validated binding. Abbreviations: RPISeq, RNA-protein interaction prediction using sequence information; 3′UTR, 3′ untranslated region; 5′UTR, 5′ untranslated region. Some elements of the schematic were generated with assistance from ChatGPT (OpenAI, GPT-5).

Model 3: CTH could potentially interact with *HIF1A* mRNA. This possibility is currently hypothesis-generating and supported primarily by computational prediction. Sequence-based prediction using RPISeq [151,152], which applies Support Vector Machine (SVM) and Random Forest (RF) classifiers trained on PRIDB datasets [153], suggests a potential interaction between CTH and *HIF1A* mRNA. As shown in Figure 8A, CTH exhibits higher predicted interaction probabilities with *HIF1A* (RF: 0.75; SVM: 0.99) than with a comparison transcript (*PTBP1*; RF: 0.60; SVM: 0.68), indicating potential transcript selectivity rather than nonspecific RNA binding; however, these differences should be interpreted only as suggestive of transcript selectivity and not as evidence of binding specificity. Region-resolved analysis across the HIF1A transcript further reveals heterogeneous interaction probabilities (Figure 8B), with stronger predicted signals in the coding sequence (RF: 0.70; SVM: 0.99) and 3′UTR (RF: 0.65; SVM: 0.99), and weaker signals in the 5′UTR (SVM: 0.38). Based on these predictions, we present a schematic hypothesis illustrating preferential interaction within the coding sequence (Figure 8C). This is intended as a testable Model 3 framework rather than evidence of validated binding. Collectively, these findings support a potential non-random, transcript- and region-specific interaction profile between CTH and *HIF1A* mRNA, warranting further experimental validation. The converging lines of evidence supporting Models 1–3, together with additional structural and non-canonical functional support, are summarized in Table 2.

Together, these models provide mechanistic hypotheses for how CTH may integrate signaling and translational control pathways to regulate gene expression programs involved in tumor progression and adaptation.

Evidence types are classified as follows: Direct experimental refers to functional studies demonstrating changes in HIF-1α protein or translation-related outputs following CTH perturbation. Experimental (indirect mechanistic) refers to pathway-level or interactome-based evidence suggesting mechanistic involvement without direct measurement of translational regulation. Computational refers to in silico analyses without experimental validation in this framework. Conceptual synthesis denotes integrative model-based interpretation derived from multiple evidence layers. “Hypothesis-generating” and “speculative” denote mechanistic interpretations not directly supported by functional or biochemical validation, and are included to reflect emerging or conceptual extensions of the model. “Limitations” indicate unresolved mechanistic gaps or lack of functional validation in CCOC. Abbreviations: CCOC, clear cell ovarian carcinoma; RBPs, RNA-binding proteins; IP-MS, immunoprecipitation coupled with mass spectrometry; RPIseq, RNA-protein interaction prediction using sequence information; CDS, coding sequence; UTR, untranslated region; IDRs, intrinsically disordered regions; WB, Western blotting.

## 9. Conclusions and Future Research Directions

Collectively, the evidence presented here supports a conceptual shift in the understanding of cystathionine γ-lyase (CTH). While traditionally viewed as a metabolic enzyme involved in cysteine biosynthesis and hydrogen sulfide (H_2_S) production, CTH may also exhibit broader, context-dependent functions extending to the regulation of mRNA translation.

This emerging perspective is supported by multiple converging features, including structural flexibility, multi-compartment localization, interaction with translation-associated proteins and RBPs, and modulation of translational signaling pathways such as PI3K/AKT/mTOR. Collectively, these observations position CTH within regulatory networks that coordinate cellular responses to metabolic and environmental stress. However, the current evidence remains largely indirect, and whether CTH directly regulates mRNA translation remains unresolved. The hypothesis that CTH contributes to translational control—particularly of transcripts such as *HIF1A*—should therefore be viewed as a testable framework rather than an established mechanism.

Future studies in CCOC cell lines, organoid systems, and other patient-derived models should therefore integrate loss- and gain-of-function genetic models of CTH (knockdown/knockout and overexpression systems) with approaches that directly interrogate the relationship between CTH and the translational machinery. Addressing these hypotheses will require systematic experimental validation using complementary approaches, including:

- RNA association assays, including CLIP-seq to assess whether CTH is associated with RNA, and RIP-qPCR to evaluate its presence in ribonucleoprotein complexes.
- Protein interactome profiling (e.g., immunoprecipitation coupled with mass spectrometry, IP-MS) to identify CTH-interacting partners, particularly components of RNA-binding and translational regulatory complexes.
- Polysome profiling to determine whether CTH alters global translational state and ribosome distribution across mRNA populations, with fraction-specific RNA analysis (qPCR or sequencing) to identify transcripts enriched in translating fractions.
- Ribosome profiling to evaluate whether CTH modulates genome-wide and transcript-specific translational efficiency at nucleotide resolution.
- Reporter assays using *HIF1A* 5′ UTR/CDS/3′ UTR or full-length constructs in the presence of recombinant or cellular CTH modulation to determine whether CTH directly regulates *HIF1A* mRNA translation, particularly given prior evidence that CTH modulates HIF-1α abundance [7], and affects the expression of HIF-1α downstream targets such as VEGF [7,37].
- Functional rescue experiments using catalytic-dead CTH mutants to distinguish enzymatic from non-enzymatic roles.

These approaches are intended to both validate and potentially falsify key components of the proposed model, enabling rigorous testing of the framework. They will also be essential to establish whether CTH functions as a direct or indirect regulator of mRNA translation and to define the molecular mechanisms involved. From a therapeutic perspective, these considerations suggest that current strategies focused solely on inhibiting CTH enzymatic activity, such as the use of competitive inhibitors like propargylglycine (PAG) [154], may not fully capture its functional roles, particularly if non-canonical interaction-mediated mechanisms are involved. In this context, our recent findings in CCOC models showed that both genetic and pharmacological targeting of CTH enhanced sensitivity to the mTOR inhibitor everolimus, with a noticeably more pronounced effect observed following genetic modulation of CTH [155]. Nevertheless, given the limited direct mechanistic evidence, such implications should be regarded as future possibilities rather than immediate therapeutic opportunities.

Although this review focuses on CCOC as a biologically informative model system, the principles outlined here may extend to other cancer types and physiological contexts in which CTH is expressed and dynamically regulated. More broadly, this work highlights a growing paradigm in which metabolic enzymes contribute to gene regulation beyond their canonical biochemical functions, revealing additional layers of post-transcriptional control linking metabolic state to selective protein synthesis.

## 10. Caveats and Alternative Mechanisms

While our findings and the available evidence support a potential role for CTH in post-transcriptional regulation, whether this extends to direct regulation of mRNA translation remains unresolved and requires experimental validation. Should such a role exist, its effects may not be restricted to translation initiation but could extend to other stages of the translational cycle. Translation is a multi-step, highly coordinated process [156] encompassing initiation, elongation, termination, and ribosome recycling. For instance, CTH activity may influence elongation rates or ribosome processivity under specific cellular contexts, including hypoxia and oxidative stress. Notably, emerging evidence links cellular redox status to ribosome pausing and codon-specific translation dynamics [157,158], raising the possibility that CTH-dependent sulfur metabolism could intersect with these processes.

In addition, CTH may influence mRNA partitioning into stress granules or processing bodies—dynamic non-membranous cytoplasmic assemblies that form under stress and function as transient storage sites for mRNAs [159]—thereby indirectly shaping translation efficiency. This possibility is supported by the close coupling of stress granule assembly to translational arrest and redox signaling [160]. These processes are particularly relevant in hypoxic and metabolic stress contexts, where both CTH expression and translational control pathways are dynamically regulated. Notably, exogenous H_2_S has been shown to promote stress granule formation and induce translational repression [161], suggesting that CTH may participate in these regulatory networks.

Finally, we cannot exclude potential crosstalk with non-canonical translation pathways, including internal ribosome entry site (IRES)-mediated initiation. These mechanisms may become especially important when canonical translation initiation is altered or restricted. Together, these considerations highlight that although our data emphasize translation as a key node of CTH function, additional mechanisms may contribute to the observed phenotypes. Dissecting these possibilities will be important for developing a more comprehensive understanding of how CTH integrates metabolic and translational control.

## Figures and Tables

**Table 1:** Reported and proposed compartment-specific functions of CTH.

Cellular Compartment	Evidence Type	Proposed/Reported Functions	Representative References
Cytoplasm	Well-established canonical function	Conversion of cystathionine to cysteine, intracellular H_2_S generation, GSH synthesis, and redox homeostasis	[92]
Nucleus	Experimental	Cysteine oxidation, histone H3 acetylation, potential chromatin remodeling and transcriptional regulation	[93]
Mitochondria	Experimental	Enhanced mitochondrial ATP production in SMCs under hypoxia, H_2_S production, and regulation of mitochondrial redox balance and bioenergetics	[94]
Plasma membrane	Experimental	Localized H_2_S generation with potential roles in membrane-associated signaling and adaptive responses to environmental stressors	[95]
Endoplasmic reticulum	Experimental	Intracellular H_2_S production, contributing to tonic vascular relaxation, and EDHF-mediated signaling	[95]
Plasma and serum	Direct biochemical measurement	H_2_S production and protection against serum deprivation and hypoxia-reoxygenation injury	[96]
Urine (extracellular exosomes)	Large-scale proteomics	Putative biomarker function?	[97]
Lysosome, Golgi, cytoskeleton, and others	Database annotations and sequence-based predictions.	To be validated and characterized	GeneCards *

H_2_S: Hydrogen sulfide; GSH: Glutathione; ATP: Adenosine tri-phosphate; SMCs: Smooth muscle cells; EDHFL: Endothelium-derived hyperpolarizing factor. * https://www.genecards.org/cgi-bin/carddisp.pl?gene=CTH, accessed on 16 May 2026).

**Table 2:** Summary of Evidence Potentially Linking CTH to HIF-1α Regulation and Translational Control.

**A. Direct HIF-1α regulatory evidence**			
**Proposed Mechanism**	**Evidence**	**Type**	**Limitation**
CTH regulates HIF-1α protein abundance under hypoxia	CTH knockout reduces HIF-1α protein levels in CCOC [7]	Direct experimental	Mechanism unresolved
CTH acts post-transcriptionally on HIF-1α in CCOC	No change in *HIF1A* mRNA despite altered HIF-1α protein levels; HIF-1α protein stability and proteasome-dependent degradation are not affected by CTH depletion [7]	Direct experimental	Does not alone establish translational regulation
**B. Signaling-mediated translational regulation**			
**Proposed Mechanism**	**Evidence**	**Type**	**Limitation**
CTH modulates PI3K/AKT/mTOR signaling affecting translation	Altered PI3K/AKT/mTOR pathway activity observed following CTH perturbation [37,40,75,124]	Experimental (indirect mechanistic)	No direct link to *HIF1A* mRNA translation in CCOC
**C. Translational machinery-associated regulation**			
**Proposed Mechanism**	**Evidence**	**Type**	**Limitation**
CTH may associate with proteins implicated in translational control in mammalian systems	Association with YTHDF1 [120], which promotes translation efficiency of target mRNAs via recruitment of eIF3 [127,128], and YWHAZ [123], which has been implicated in selective mRNA translation and PI3K/AKT/mTOR pathway activation [123,129,130]	Experimental/ Interactome (indirect mechanistic)	Functional relevance in CCOC not validated
Cross-species association with translation initiation machinery	Association with RpS2 in *Drosophila melanogaster* and *Saccharomyces cerevisiae* [125], as well as with eIF3j in *Drosophila melanogaster* [126]	Experimental/Interactome (indirect mechanistic)	Conservation and functional relevance in mammalian and CCOC systems not confirmed
**D. RNA-binding-related mechanisms**			
**Proposed Mechanism**	**Evidence**	**Type**	**Limitation**
CTH interacts with RBPs implicated in *HIF1A* mRNA translation regulation	ELAVL1 and PTBP1 identified by IP-MS; ELAVL1 association confirmed by WB [77]	Experimental/ Interactome (indirect mechanistic)	No functional validation in translational control
CTH may directly bind *HIF1A* mRNA	RPISeq prediction scores suggest possible interaction [151,152]	Computational (speculative)	No experimental RNA-binding validation
Region-specific CTH-*HIF1A* interaction potential	Differential RPISeq scores across CDS/UTR regions	Computational (speculative)	No experimental validation
**E. Structural and non-canonical functional support**			
**Proposed Mechanism**	**Evidence**	**Type**	**Limitation**
CTH contains IDRs and shows multi-compartment localization patterns	Multiple lines of structural and computational evidence of intrinsic disorder and structural flexibility [91,108,109], together with experimental and large-scale proteomics/interactome datasets, indicate distribution across cytosolic and multiple subcellular compartments [93,94,95,96,97,98]	Experimental & Computational (structural and localization evidence)	IDRs do not inherently confer RNA-binding function and localization does not confirm functional RNA-binding or translational activity
CTH functions extend beyond H_2_S metabolism	CTH exerts H_2_S-independent functions [36,110], H_2_S donors did not rescue CTH-loss phenotypes in some models [41]; preserved sulfide/persulfide levels despite disruption of canonical sulfur-metabolizing enzymes in mice [70] and in a patient with profound CTH deficiency [69]	Experimental (H_2_S-independent functional evidence)	Indirect link to translation
**F. Integrated/hypothesis-generating models**			
**Proposed Mechanism**	**Evidence**	**Type**	**Limitation**
CTH may regulate translation through a multi-layered molecular network	Integration of signaling, interactome, phenotypic, and structural evidence	Conceptual synthesis (hypothesis-generating model)	Requires mechanistic validation

## Data Availability

No new datasets were generated or analyzed in this study. All data used are publicly available from the cited sources.

## Abbreviations

4E-BPs	Eukaryotic translation initiation factor 4E-binding proteins
ADK	Adenylate kinase
AKT	Protein Kinase B
AP-MS	Affinity purification-mass spectrometry
ARID1A	AT-rich interactive domain 1A
ASS1	Argininosuccinate synthase 1
ATF 4	Activating transcription factor 4
CARS	Cysteinyl-tRNA synthetase
CAT	Cysteine aminotransferase
CBS	Cystathionine β-synthase
CCOC	Clear cell ovarian carcinoma
CDS	Coding sequence
CLIP-seq	Cross-linking immunoprecipitation sequencing
CREB	cAMP response element-binding protein
CRY1	Cryptochrome 1
CTH	Cystathionine γ-lyase
CUL4A	Cullin-4A
D2P2	Database of Disordered Protein Predictions
DMOG	Dimethyloxalylglycine
eIF3	Eukaryotic translation initiation factor 3
eIF3j	Eukaryotic translation initiation factor 3, subunit J
eIF4B	Eukaryotic translation initiation factor 4B
eIF4E	Eukaryotic translation initiation factor 4E
eIF4F	Eukaryotic translation initiation factor 4F
ELAVL1	ELAV like RNA binding protein 1
Elk1	ETS like-1
ER	Endoplasmic reticulum
FOXD3	Forkhead box D3
FXR	Farnesoid X receptor
GAPDH	Glyceraldehyde 3-phosphate dehydrogenase
GCLC	Glutamate–cysteine ligase catalytic subunit
GH	Growth hormone
GSH	Glutathione
GSH-MEE	Glutathione monoethyl ester
H_2_S	Hydrogen sulfide
HIF-1α	Hypoxia-inducible factor-1α
HuR	Human antigen R
IDRs	Intrinsically disordered regions
IRES	Internal ribosome entry site
KH	K homology
KO	Knockout
miRs	MicroRNAs
MoRFs	Molecular recognition features
MPST	3-mercaptopyruvate sulfurtransferase
mRNA	Messenger ribonucleic acid
mTOR	Mechanistic target of rapamycin
NF-κB	Nuclear factor kappa B
Nrf2	Nuclear factor erythroid 2–related factor 2
NSCLC	Non-small cell lung cancer
PAG	Propargylglycine
PARK2	Parkinson disease 2/Parkin
PDHA1	Pyruvate dehydrogenase E1 subunit alpha 1
PI3K	Phosphatidylinositol 3-kinase
PKM2	Pyruvate kinase M2
PLP	Pyridoxal 5′-phosphate
PPIs	Protein–protein interactions
PTBP1	Polypyrimidine tract binding protein 1
PTMs	Post-translational modifications
RBPs	RNA-binding proteins
RF	Random Forest
RIP-qPCR	RNA immunoprecipitation-quantitative polymerase chain reaction
RNA	Ribonucleic acid
ROS	Reactive oxygen species
PRIDB	Protein-RNA Interface Database
RPISeq	RNA-protein interaction prediction using sequence information
RpS2	Ribosomal protein S2
RRMs	RNA recognition motifs
S6K	Ribosomal protein S6 kinase
SAM	S-adenosylmethionine
SCD1	Stearoyl-CoA desaturase
SCOP	Structural Classification of Proteins
SDC1	Syndecan 1
SGs	Stress granules
SP1	Specificity protein 1
SREBP1	Sterol regulatory element-binding protein 1
SVM	Support Vector Machine
TH	Thyroid hormone
TNBC	Triple-negative breast cancer
TSS	Transsulfuration
UTR	Untranslated region
VEGF	Vascular endothelial growth factor
WB	Western blotting
YAP	Yes-associated protein
YTHDF1	YTH N6-methyladenosine RNA-binding protein 1
YWHAZ	Tyrosine 3-Monooxygenase/Tryptophan 5-Monooxygenase Activation Protein Zeta
